# Comparison of early risk factors between healthy siblings and subjects with schizophrenia and bipolar disorder

**DOI:** 10.3389/fpsyt.2024.1374216

**Published:** 2024-04-30

**Authors:** Rosany Guterrez Nunes, Carolina Gomes Carrilho, Gilberto Sousa Alves, Dolores Malaspina, Jeffrey Paul Kahn, Antonio Egidio Nardi, André Barciela Veras

**Affiliations:** ^1^ Postgraduate Program in Health Psychology of the Dom Bosco Catholic University of Campo Grande - MS, Campo Grande, MS, Brazil; ^2^ Medical School of the Federal University of Maranhão, São Luis, MA, Brazil; ^3^ Departments of Psychiatry, Neuroscience and Genetics, Icahn School of Medicine at Mt. Sinai Medical Center, New York, NY, United States; ^4^ Department of Psychiatry, Weill-Cornell Medical College, New York, NY, United States; ^5^ Laboratory of Panic and Respiration, Institute of Psychiatry of the Federal University of Rio de Janeiro, Rio de Janeiro, RJ, Brazil

**Keywords:** gestational risk factors, early trauma, obstetric history, schizophrenia, bipolar disorder

## Abstract

**Introduction:**

The following work aims to compare the types and magnitude of risk events in patients with Schizophrenia and Bipolar Disorder and each of those groups with of a group of healthy siblings, exploring differences and similarities of the two psychotic disorders.

**Methods:**

Retrospective interviews were conducted with 20 families to investigate maternal and obstetric health, social support and the presence of early trauma for the affected family members and healthy siblings. Mothers were interviewed with the Prenatal Psychosocial Profile and each family participant was assessed with the Early Trauma Inventory, Screening Questionnaire of the Genomic Psychiatry Cohort and the Diagnostic Interview for Psychosis and Affective Disorders.

**Results:**

Obstetric and gestational history, pregnancy weight changes and early trauma were associated with offspring’s mental illness, including statistically significant findings for complications of pregnancy, pregnancy weight changes, general trauma, physical punishment and emotional abuse.

**Conclusion:**

These findings highlight the different risk factor exposures that occur within a family, which may increase the risk for severe mental illness.

## Introduction

Schizophrenia is a severe psychiatric disorder that presents with psychotic symptoms in late adolescence or young adulthood. Affecting approximately 1% of the worldwide population, its symptoms include cognitive, behavioural and emotional deficits, that substantially impact functioning (1). Bipolar disorder can also present with psychosis, but is marked by extreme mood swings that range from extremely elevated and expansive mania to periods of intense sadness, agitation and even suicide attempts (2).

Both schizophrenia and bipolar disorder have complex genetic influences whose underpinnings include genetic, environmental and psychosocial factors (1, 3). Both conditions have developmental components, and the risk-related exposures include events and exposures in the pre and perinatal period, and early childhood trauma (4). Exposures can alter the expression of genes through epigenetic mechanisms, producing lasting changes in physiology and brain function, which can interact with genetic vulnerability to increase the risk for mental disorder (5).

Our initial hypothesis was that adverse events could trigger such disorders and thus that affected offspring would have more exposures than their healthy siblings and that Schizophrenia and Bipolar Disorder share similar types of risk events but may differ in term of magnitude and also show qualitative differences. The current study was a case-control investigation on exposures to risk events, including maternal health in pregnancy, obstetric complications, psychological and social health, and early trauma (general trauma, physical abuse, emotional abuse and sexual abuse) simultaneously comparing offspring with Schizophrenia and Bipolar Disorder to their own healthy siblings.

## Methods

A case-control study was conducted in a sample of 20 families (N = 20 mothers; N = 20 offspring with schizophrenia (N = 12) or bipolar I disorder (N = 08) and N = 20 healthy control offspring). Recruitment was done by convenience with patients awaiting outpatient medical consultation at two Psychosocial Care Centres (PCC) for mental health in Campo Grande, Mato Grosso do Sul State – Brazil. Interviews lasted an average of 40 minutes and were carried out at both PCCs, as well as at the individual’s home when appropriate.

Inclusion criteria for offspring included subjects from both sexes (male/female), older than 18 years old, with a diagnosis of schizophrenia or bipolar disorder I according to the diagnostic criteria of the Diagnostic and Statistical Manual of Mental Health – 5 (2), who were in outpatient treatment at a PCC in Brazil. All subjects signed an Informed Consent Form (ICF). The study was approved by the local Ethics Committee (n° 2.797.686) according to the protocol of Helsinki, and all patients provided informed consent after receiving complete explanation of the study protocol.

The affected offspring in each family (20 cases) were interviewed with the Screening Questionnaire for the Genomic Psychiatry Cohort (Portuguese Version) to identify symptoms of schizophrenia, mania, depression, substance use and health history. We also used the Diagnostic Interview for Psychosis and Affective Disorders (Di-PAD) (6), which uses diagnostic criteria for schizophrenia and bipolar disorder and sociodemographic data and questions to identify the presence of depression, psychotic symptoms and mania. And the Early Trauma Inventory Self Report–Short Form (ETISR-SF) (7), a 27 item scale assessing the occurrence of early trauma (general trauma, physical abuse, emotional abuse and sexual abuse) after 18 years-old. The Screening Questionnaire for the Genomic Psychiatric (Portuguese Version) and the Early Trauma Inventory were also administered to the healthy siblings. Comparison was made between siblings, therefore, individuals with schizophrenia were compared with their own siblings, and individuals with bipolar disorder were also compared with their own siblings.

Mothers were interviewed with the Prenatal Psychosocial Profile, translated for use in Brazil in 2007 (8), in reference to her child participants. Each affected subject had one sibling from the same mother and father selected to participate as a control. The Prenatal Psychosocial Profile is a Likert scale with 44 items that elicit the presence of stress (day-to-day stress, financial concerns, transportation, bills to pay, housing problems, family problems, the loss of a loved one, current pregnancy, situations of abuse or violence, alcohol or drug problems), social support (financial and emotional support from their partner and other people) and self-esteem in women during the pregnancy of their healthy and mentally ill offspring.

The comparisons in this study were between cases and controls. Complex analysis such as clustering or pairing was not considered because of the characteristic of a pilot-study with a small sample. Data analysis was conducted using Stata version 12.0. Continuous variables were tested for normality using the Shapiro-Wilk and Kolmorogov-Smirnov tests. Categorical variables were analysed using Chi-square tests, and continuous variables with Student’s t-test. Significance was set at *p* < 0.05.

## Results

### Obstetric complications – offspring with schizophrenia and their healthy siblings

There was no significant statistical difference when comparing to their healthy siblings in adverse obstetric events (complications of pregnancy: viral infections during the second trimester of pregnancy, recurrent urinary tract infections with hospital admissions, depressive disorders with ideation and suicide attempt during the second trimester of pregnancy, and inadequate maternal weight gain due to insufficient supply of caloric and protein intake). There was a trend towards significance in the variables investigated (*p* = 0.07), but it was not considered a significant difference for individuals with schizophrenia and their healthy siblings, thus suggesting that the number of prenatal consultations did not influence the event investigated.

An analysis (**Table 1**) was also performed in relation to prenatal consultations (*p value* = 1.0), which showed no significant difference when comparing to their healthy siblings, including such items as type of delivery (*p value* = 0.6), therefore, caesarean versus vaginal delivery does not predict the presence or absence of a disorder among siblings in the present sample. Regarding term versus preterm gestational age (*p value* = 0.3) there was no significant statistical difference in subjects with schizophrenia.

**Table 1:** Correlation of obstetric data of offspring with Schizophrenia and their healthy siblings.

			Group				
			Schizophrenia	Control			P value
Number of prenatal consultations			3.25±2.9	3,58±3,3		*m±sd*	0.7
Prenatal consultation	Yes		7 (50%)	7 (50%)			
	No		5 (50%)	5 (50%)		N (%)	1.0
Delivery							
	Vaginal		8 (53,33%)	7 (46,67%)			
	Caesarean		4 (44,44%)	5 (55,56%)		N (%)	0.6
Gestational age							
	Term		11 (47,83%)	12 (52,17%)			
	Preterm		1 (100%)	0 (0%)		N (%)	0.3
Complications of pregnancy							
	Yes		3 (100%)	0 (0%)			
	No		9 (42,86%)	12 (57,14%)		N (%)	0.06
Labour complications							
	Yes		1 (50%)	1 (50%)			
	No		11 (50%)	11 (50%)		N (%)	1.0
Medication use during pregnancy							
	Yes		8 (53,33%)	7 (46,67%)			
	No		4 (44,44%)	5 (55,56%)		N (%)	0.6
Prenatal in other pregnancies							
	Yes		8 (47,06 %)	9 (52,94%)			
	No		4 (57,14%)	3 (42,86%)		N (%)	0.6
Planned pregnancy							
	Yes		1 (50%)	1 (50%)			
	No		11 (50%)	11 (50%)		N (%)	1.0
Abortion attempt							
	Yes		0 (0%)	0 (0%)			
	No		12 (50%)	12 (50%)		N (%)	1.0
Pregnancy weight changes							
	Yes		3 (25%)	0 (75%)			
	No		9 (0%)	12 (10%)		N (%)	0.06
Blood pressure problems during pregnancy							
	Yes		1 (100 %)	0 (0%)			
	No		11 (47,83%)	12 (52,17%)		N (%)	0.3
Complications of previous pregnancies							
	Yes		5 (62,50%)	3 (37,50%)			
	No		7 (43,75%)	9 (56,25%)		N (%)	0.3

Obs: m±sd (mean and standard deviation) used for t test and N (%) (total value and percentage of events per group) used for Pearson’s chi-square and Fisher’s exact test correlation.

There was a trend toward statistical significance in the complications of pregnancy variable (*p value* = 0.06), which may indicate that in all of the individuals with pregnancy complications, three had Schizophrenia, thus hinting possible significance, since there were no such complications among healthy siblings. The following variables were tested: complications of pregnancy (*p value* = 1.0), medication use during pregnancy (*p value* = 0.6), prenatal in other pregnancies (*p value* = 0.6), planned pregnancy (*p value* = 1.0) and abortion attempt (*p value* = 1.0). Those variables showed no significant statistical difference for individuals with mental illness and their siblings.

In contrast, there was a trend toward statistical significance in pregnancy weight changes for both individuals with mental illness and their siblings (*p value* = 0.06). Pregnancy weight changes were more present during the pregnancies of individuals with schizophrenia. However, blood pressure problems during pregnancy (*p value* = 0.3) and complications of previous pregnancies (*p value* = 0.3) showed no statistical significance when comparing to their healthy siblings.

### Obstetric complications – offspring with bipolar disorder and their healthy siblings

There was no significant statistical difference between mean values of the variables investigated (*p value* = 0.3) for individuals with bipolar disorder and their healthy siblings, implying that the number of prenatal consultations had no influence in the occurrence of the event investigated.

No significant correlation was observed between the following variables: prenatal consultation (*p value* = 0.6) and type of delivery (*p value* = 0.5). Regarding the gestational age at term or premature birth, no significant difference was found in subjects with bipolar disorder when comparing to their healthy siblings.

Nonetheless, a trend towards significance was found for complications of pregnancy (*p value* = 0.05). The three individuals with pregnancy complications all had bipolar disorder in adulthood, in contrast with no such complications in healthy siblings. No significant statistical differences were found in the following variables: complications of pregnancy (*p value* = 0.1), medication use during pregnancy (*p value* = 1.0), prenatal in other pregnancies (*p value* = 0.2), planned pregnancy and abortion attempt (*p value* = 0.2), blood pressure problems during pregnancy (*p value* = 0.31) and complications of previous pregnancy (*p value* = 0.1).

On the contrary, pregnancy weight changes showed significant statistical difference in subjects with schizophrenia in comparison with their healthy siblings (*p value* = 0.05). This variable was more frequently present in the pregnancy of offspring with bipolar disorder 100% (*N = 3*) (**Table 2**).

**Table 2:** Correlation of obstetric data of offspring with bipolar disorder and their healthy siblings.

		Group			
		BD	Control		P value
Number of prenatal consultations		3.5±3.2	4.3±3.6	*m±dp*	0.3
Prenatal consultation					
	Yes	4 (44,44%)	5 (55,56 %)		
	No	4 (57,14%)	3 (42,86%)	N (%)	0.6
Delivery					
	Vaginal	6 (54,55%)	5 (45,45 %)		
	Caesarean	2 (40 %)	3 (60%)	N (%)	0.5
Gestational age					
	Term	8 (50%)	8 (50%)		
	Preterm	0 (0%)	0 (0%)	N (%)	NS
Complications of pregnancy					
	Yes	3 (100%)	0 (0%)		
	No	5 (38,46%)	8 (61,54%)	N (%)	0.05
Labour complications					
	Yes	2 (100%)	0 (0%)		
	No	6 (42,86%)	8 (57,14%)	N (%)	0.1
Medication use during pregnancy					
	Yes	4 (50 %)	4 (50%)		
	No	4 (50%)	4 (50%)	N (%)	1.0
Prenatal in other pregnancies					
	Yes	5 (41,67%)	7 (58,33%)		
	No	3 (75%)	1 (25%)	N (%)	0.2
Planned pregnancy					
	Yes	0 (0%)	0 (0%)		
	No	8 (50%)	8 (50%)	N (%)	NS
Abortion attempt					
	Yes	1 (100%)	0 (0%)		
	No	7 (46,67%)	8 (53,33%)	N (%)	0.3
Pregnancy weight changes					
	Yes	3 (100%)	0 (0%)		
	No	5 (38,46%)	8 (61,54%)	N (%)	0.05*
Pregnancy blood pressure problems					
	Yes	2 (100 %)	0 (0%)		
	No	6 (42,86%)	8 (57,14%)	N (%)	0.1
Complications of previous pregnancies					
	Yes	2 (100 %)	0 (0%)		
	No	6 (42,86%)	8 (57,14%)	N (%)	0.1

Obs: m±sd (mean and standard deviation) used for t test and N (%) (total value and percentage of events per group) used for Pearson’s chi-square and Fisher’s exact test correlation.

### Early trauma – offspring with schizophrenia and their healthy siblings

**Figure 1:**
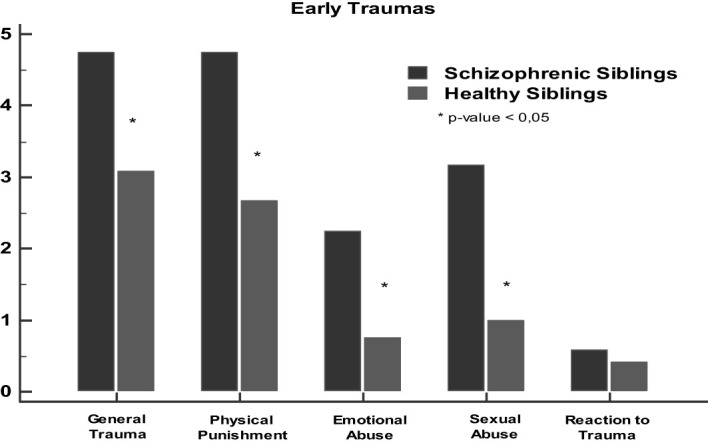
Correlation of early trauma data (ETISR-SF) between offspring with schizophrenia and their healthy siblings. *p < 0.05.

Affected offspring with schizophrenia had a higher score of general trauma (4.7 ± 2.1) in comparison with their healthy siblings (3.08 ± 2.02). Compared to their siblings, patients with schizophrenia had higher scores for physical punishment (*p value* = 0.006) (4.75 ± 2.1 vs 2.6 ± 1.5), for emotional abuse (*p value* = 0.009), (2.6 ± 1.6 vs 0.75 ± 1.2), and for sexual abuse (*p value* = 0.006), (3.1 ± 2.1 vs 1 ± 2.2). Lastly, there was no significant statistical difference in reaction to trauma (fear or depersonalisation) (*p value* = 0.3) among both groups of subjects when comparing to their healthy siblings (**Figure 1**).

### Early trauma – offspring with bipolar disorder and their healthy siblings

**Figure 2:**
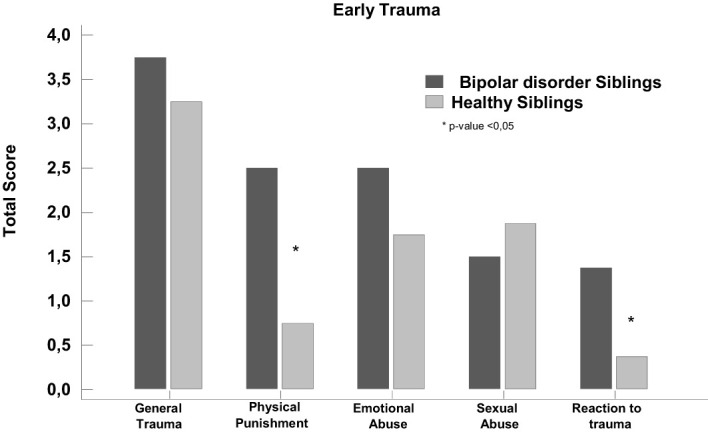
Correlation of early trauma data (ETISR-SF) between offspring with bipolar disorder and their healthy siblings. *p < 0.05.

For comparison between early trauma data (ETISR-SF) in subjects with bipolar disorder and their healthy siblings (**Figure 2**), total scores and subscales were analysed.

No significant statistical difference was found for general trauma (*p value* = 0.3), but a significant difference was found for physical punishment (*p value* = 0.008), which points to a greater presence of physical punishment in individuals with bipolar disorder (3.75 ± 1.5) compared to their healthy siblings (3.25 ± 2.08). No significant statistical difference was found for emotional abuse (*p value* = 1.0), sexual abuse (*p value* = 0.3), reaction to trauma (*p value* = 0.0009), although subjects with bipolar disorder showed higher scores for reaction to trauma (1.3 ± 0.5) when compared to their healthy siblings (0.3 ± 0.5) (**Figure 2**).

## Discussion

The present investigation revealed that complications in pregnancy and childbirth are similar in rates in the offspring with schizophrenia and bipolar disorder, and more frequent in the pregnancies of these individuals than in those of their unaffected siblings.

The significance found in the variables complication of pregnancy and pregnancy weight changes are in line with previous study (9), which reported pregnancy and obstetric complications, maternal weight change (malnutrition or overweight) and maternal diseases in pregnancy are associated with schizophrenia onset, as well as nutritional and multivitamin deficiencies (iron, vitamin D, folic acid, iodine). Obstetric occurrences, are also important risk factors, either due to physiological impairment or foetal neurodevelopment, which ultimately compromise the individual’s mental health in early life (4, 10).

General trauma was also more prevalent in the mentally ill group, with a significant statistical difference (*p value* = 0.02). In trauma for physical punishment (*p value* = 0.0004) and emotional abuse (*p value* = 0.003) the score was also higher in mentally ill individuals. Our findings are in accordance with researches pointing out that traumatic events in childhood may favour mental disorders such as schizophrenia and bipolar disorder because of genetic interaction with environmental exposure (11). However, those with later illness also could have greater early behavioral problems, leading to negative parental conflicts.

In the group of offspring with mental illness, there were more reports of events of general and emotional trauma and physical punishment, which may have contributed to the onset of the disorders in this group. Our findings are in agreement with the scientific literature that points to the registry of trauma, abuses, abandonment and neglect in childhood as variables present when diagnosing Bipolar Disorder and schizophrenia (11, 12). And these gene-environment interactions (genetic susceptibility), coupled with childhood traumata, in the presence of bipolar disorder, are presented in a much more serious way, with the clinical expression of the disorder for suicidal behaviour (12).

Finally, results of the data collected revealed that variables related to the gestational age and medical history of mothers were important (complications of pregnancy and pregnancy weight changes), as risk factors associated with the events of early trauma, which were relevant in the group with mental illness. Our findings confirm and are in accordance with scientific literature, that points to schizophrenia and bipolar disorder as a consequence of the association between adverse events and biopsychosocial risk factors during pregnancy and childhood of the investigated individuals.

Some limitations of our study deserve further comment. Firstly, the cross-sectional retrospective nature of our study, thereby precluding any firm conclusion on the cause-effect relation between emotional trauma and clinical features of schizophrenia. In addition, there is a potential recall bias in self-reported data of the affected subjects, once patients are affected by severe mental health suffering in comparison to controls. Secondly, the lack of primary sources of medical information, e.g., obstetric and paediatric records and the potential influence of memory bias on the data collected. Thirdly, our study was conducted with a small sample of patients with schizophrenia (N = 12) and bipolar I disorder (N = 8). Although it is not a homogeneous sample, both share psychosis and risk factors, as observed in our study. However, the size of the sample could not allow a generalization of the comparison between these two disorders. A higher-powered study could provide stronger statistical support for these findings. Despite these constraints, data was consistently confirmed and re-checked to ensure its reliability and all interviews were carried out with standardized and valid instruments for the Brazilian environment, therefore providing consistent findings.

In our study, the use of sibling controls may have contributed to better estimate the importance of early risk factors in the development of schizophrenia and bipolar disorder. However, adding another set of patients not related to siblings for an additional comparison with the groups of this study would have been helped to clarify even more questions about the importance of the researched risk factors. Future researches may contribute with this methodological improvement.

## Data availability statement

The raw data supporting the conclusions of this article will be made available by the authors, without undue reservation.

## Ethics statement

The studies involving humans were approved by CEP Universidade Católica Dom Bosco. The studies were conducted in accordance with the local legislation and institutional requirements. The participants provided their written informed consent to participate in this study.

## Author contributions

RN: Writing – review & editing, Writing – original draft. CC: Writing – original draft. GA: Writing – review & editing. DM: Writing – review & editing. JK: Writing – review & editing. AN: Writing – review & editing. AV: Writing – review & editing.
